# LTA + 252A > G polymorphism is associated with risk of nasal NK/T-cell lymphoma in a Chinese population: a case-control study

**DOI:** 10.1186/s12885-015-1506-4

**Published:** 2015-06-25

**Authors:** Sensen Cheng, Jianzhong Li, Wenjian Liu, Chengxiang Liu, Lei Su, Xiuchun Liu, Liangjun Guo, Yuan Ma, Bao Song, Jie Liu

**Affiliations:** 1 Department of Oncology, Shandong Cancer Hospital and Institute, Shandong Academy of Medical Sciences, 440 Jiyan Road, Jinan, 250117 China; 2 School of Medicine and Life Sciences, University of Jinan, Shandong Academy of Medical Sciences, Jinan, China; 3 Department of Oncology, General Hospital of Jinan Iron and Steel Group Limited Company, Jinan, China; 4 Department of Oncology, Affiliated Hospital of Taishan Medical College, Taian, China; 5 Department of Oncology, Zhangqiu People’s Hospital of Shandong Province, Jinan, China; 6 Basic Laboratory, Shandong Cancer Hospital and Institute, Shandong Academy of Medical Sciences, 440 Jiyan Road, Jinan, 250117 China

**Keywords:** NK/T-cell lymphoma, Single nucleotide polymorphisms (SNP), Lymphotoxin-α

## Abstract

**Background:**

Nasal NK/T-cell lymphoma is a rare type of lymphoma in Caucasian individuals, but is relatively common in Asian populations. Genetic variants in immune and inflammatory response genes may thus be associated with the risk of developing lymphoma. Here, we investigated the association between immuno-modulatory gene polymorphisms and risk for nasal NK/T-cell lymphoma in a Chinese population.

**Methods:**

Analysis of 12 single nucleotide polymorphisms (SNPs) in IL-10, TNF-α, lymphotoxin-α (LTA), and CTLA-4 genes was performed for 125 patients with NK/T-cell lymphoma and 300 healthy controls by PCR-ligase detection reactions.

**Results:**

The LTA +252 GA + AA genotypes were associated with increased risk for NK/T-cell lymphoma (OR = 2.96, 95 % CI = 1.42–6.19, P = 0.004 for GA + AA genotype). Haplotype C-G-G-A (TNF-α -857, -308, −238 and LTA +252) also conferred an increased risk (OR = 1.52, 95 % CI = 1.14–2.06, P = 0.005). Additionally, the LTA +252 GA + AA genotype was associated with an even higher risk in populations positive for Epstein–Barr virus (OR = 5.20, 95 % CI = 1.22–23.41, P = 0.03 for the GA + AA genotype).

**Conclusions:**

Our data suggest that the LTA +252 A > G polymorphism is associated with the risk of developing NK/T-cell lymphoma, especially for Epstein–Barr virus-positive NK/T-cell lymphoma in the Chinese population.

## Background

NK/T-cell lymphoma is an aggressive type of cancer that attacks natural killer (NK) and/or T-cells, which are key immune cells that fight viruses, bacteria, and tumor cells. This disease is also known as nasal NK lymphoma, angiocentric lymphoma, or extranodal NK cell lymphoma. Histological features of this lymphoma are vessel-centered lesions, and extensive lymphoma infiltration of blood vessels, which results in notable ischemic necrosis of normal and neoplastic tissues [1, 2]. NK/T-cell lymphoma is relatively uncommon and accounts for less than 1 % of lymphomas in Europe and North America. However, it is relatively common in Asia and Latin America, and in China and Japan it constitutes 6–10 % of all lymphomas [1–3]. The etiology of NK/T-cell lymphoma is complicated and poorly understood, but studies suggest that Epstein–Barr virus (EBV), ethnicity, and geographic factors contribute to the etiology of this disorder [2–4], in addition to other factors that may be worthy of exploration.

There is strong evidence that altered immunological function entails an increased risk for lymphoma. Immune and inflammatory response genes are the fundamental messengers of adaptive immunity, which regulate the growth of lymphoid tissue and immune system function. Genetic polymorphisms of several immunity genes were reported to be associated with non-Hodgkin lymphoma (NHL) and its major subtypes [5–12]. For this reason, investigation of immune related genetic markers and NK/T-cell lymphoma is desirable.

Interleukin-10 (IL-10) and tumor necrosis factor (TNF) are key cytokines that have been intensively investigated in autoimmune diseases and malignancies. Both are important regulators for the Th1/Th2 balance, apoptosis, and regulation of inflammation. IL-10 knockout mouse models showed that this cytokine affects B-cell lymphomagenesis either indirectly or directly [13]. The TNF family consists of 19 members that mediate diverse biological functions in a variety of cellular systems, and TNF-α and lymphotoxin-α (LTA, also named TNF-β) are two important members of the TNF family. TNF-α is produced primarily by activation of monocyte/macrophages and LTA by lymphocytes and NK cells. Even though they are produced by different cells, their biological effect is similar. In vitro studies show that TNF-α or LTA play an important role in killing infected or tumor cells by activated macrophages and cytotoxic T cells [14]. However, the expression of endogenous TNF resists the cytotoxicity of exogenous TNF to kill tumor cells [14]. Notably, TNF may stimulate endogenous tumor promoters. Thus, it is thought to be associated with malignant tumors. A number of single nucleotide polymorphisms (SNPs) have been identified in these gene regions. Among them, IL-10 -3575 A > T and TNF-α-308 G > A SNPs were reported to be associated with NHL, especially in diffuse large B-cell lymphoma (DLBCL) by several different groups [9, 11, 12].

In addition to IL-10 and TNF, cytotoxic T lymphocyte antigen 4 (CTLA-4), a member of the immunoglobulin superfamily that is expressed mainly on activated T cells, plays a critical role in the suppression of T-cell proliferation and activation. The inhibitory role of CTLA-4 in maintaining homeostasis of inflammatory and immune reactions makes it a potential candidate gene for determining the genetic predisposition of infectious and autoimmune diseases. CTLA-4-deficient mice develop lymphoproliferative disorders characterized by polyclonal T-cell proliferation and early lethality [15]. Furthermore, CTLA-4 polymorphisms -318 C > T, +49 A > G, and CT60 A > G are associated with susceptibility to autoimmune disorders [16]. Recent reports also revealed that CTLA4 polymorphisms have a role in the occurrence of multiple myeloma [17] and NHL [18].

Because NK/T-cell lymphoma is a rare subtype of NHL worldwide, very little work has been done to understand its pathogenesis, and, to the best of our knowledge, no study has reported the genetic risk factors for this special type of NHL. In this case-control study, we investigated the association between several genetic variants of immunoregulatory genes (IL-10, TNF/LTA, and CTLA-4) and risk for nasal NK/T-cell lymphoma in a Chinese population.

## Methods

### Study subjects

This study included 125 cases that were newly diagnosed as nasal type NK/T-cell lymphoma and recruited from the following institutions: Shandong Cancer Hospital and Institute, Shandong University Qilu Hospital, Shandong Provincial Hospital, Jinan Fourth People's Hospital, and the Affiliated Hospital of Taishan Medical University. Subjects were recruited between January 2006 and December 2011. All diagnosed patients met the World Health Organization (WHO) Classification of Tumors of the Hematopoietic and Lymphoid Tissues (2008) diagnostic criteria. The pathological classification was determined based on tissue sections with haematoxylin-eosin staining and immunohistochemistry as well as clinical characteristics. The immune phenotype markers included CD2, CD3, CD56, CD45R0, TIA-1, granzyme B, LCA, and EBV status. Over the same period, 300 controls were accrued from healthy volunteers who visited the general health check-up division or patients with non-cancer diagnoses at Shandong Cancer Hospital and Institute, Shandong University Qilu Hospital, and Shandong Provincial Hospital. Controls did not have malignancy or any autoimmune or immune-mediated diseases. Randomly selected controls were matched to the cases by age (±5 years) and gender. All subjects were Han Chinese. At recruitment, informed consent was obtained from each subject. This study was approved by the Institutional Review Board of the Shandong Cancer Hospital and Institute. Institutional Review Board approval has been obtained from all study sites.

All study participants provided 2 ml of peripheral blood. Additionally, immunoglobulin-G antibodies to EBV-VCA (R-Biopharm AG, Darmstadt, Germany) were confirmed by serology testing using a standard enzyme-linked immunosorbent assay for both case and control subjects.

### Genotyping

Gene names, chromosomal location, and the SNP database IDs used for genotyping are listed in Table 1. Genotyping was carried out, blinded to case-control status, by the Shanghai Biowing Applied Biotechnology Co., Ltd. (Shanghai, China) using ligase detection reactions (LDR). Target DNA sequences were amplified using a multiplex PCR method and ligation reactions for each subject were carried out in a final volume of 10 μL containing 1 μL of 10× buffer, 100 ng of multi-PCR product, 1 pmol of each discriminating oligo, 1 pmol of each common probe, and 2 U of Taq DNA ligase (New England Biolabs, Beverly, MA, USA). The LDR parameters were as follows: 94 °C for 2 min, 35 cycles at 94 °C for 30 s, and 50 °C for 2 min. Following the LDR reaction, 1 μL of reaction product was mixed with 1-μL ROX and 1-μL loading buffer and the mixture analyzed with an ABI Prism 373 DNA Sequencer (Applied Biosystems, Foster City, CA, USA). To confirm genotyping results, 10 % of representative PCR products were examined by DNA sequencing in an ABI Prism 310 Sequence (Applied Biosystems). Results between PCR-LDR and DNA sequencing analysis were 100 % concordant.

**Table 1:** Genes and single nucleotide polymorphisms (SNPs) that were evaluated

Gene names	Description	Chromosome location	SNP rsID	Polymorphism
IL-10	Interleukin-10	1q31-q32	rs1800871	-819C > T
			rs1800872	-592C > A
			rs1800896	-1082A > G
			rs1800890	-3575 T > A
TNF-α	Tumor necrosis factor-α	6p21.3	rs1799724	-857C > T
			rs1800629	-308G > A
			rs361525	-238G > A
LTA	Lymphotoxin-alpha	6p21.3	rs909253	252A > G
CTLA4	Cytotoxic T-lymphocyte-associated 4	2q33	rs4553808	-1661A > G
			rs5742909	-318C > T
			rs231775	+49A > G
			rs3087243	CT60A > G

### Statistical analyses

Statistical analyses were performed using SAS version 9.2 (SAS Institute, Cary, NC, USA). Deviation from Hardy–Weinberg equilibrium was tested using a *χ*2 test for goodness of fit. The genotype and allele frequencies of the polymorphisms in the patient and control group were compared using a *χ*^2^ test and odds ratios (OR); 95 % confidence intervals (CIs) calculated to assess the relative risk conferred by a particular allele and genotype, adjusted for age and sex. The Benjamini–Hochberg method was used to determine the false positive discovery rate from multiple testing. Data were further stratified by EBV infection or genotypes to evaluate stratum variable related ORs. The linkage disequilibrium of the polymorphic loci and haplotypes were analyzed using SHEsis software, available from Bio-X Inc., Shanghai, China). Statistical significance was set at *P* < 0.05.

## Results

### Characteristics of NK/T lymphoma patients and controls

The demographic characteristics of cases and controls are listed in Table 2. There were no statistical differences in the age and sex distributions between cases and controls. According to the Ann Arbor–Cotswolds staging system, 63 NK/T-cell lymphoma patients were classified as stage I_E_, 38 as stage II_E_, 15 as stage III_E_, and 9 as stage IV_E_. Approximately 78.4 % (98/125) of all NK/T lymphoma cases were nasal cavity and nasopharynx, others (21.6 %, 27/125) were concentrated in the palate, oropharynx, tonsils, skin, and gastrointestinal tract.

**Table 2:** Demographic characteristics of patients with NK/T cell lymphoma and healthy controls

Characteristic	Cases (n = 125)	Controls (n = 300)	OR (95 % CI)	*P* value
*Age, years*				
Mean	43.0 ± 15.0	44.9 ± 14.8		0.569
*Sex*				
Male	83	198	1	
Female	42	102	0.98(0.63-1.53)	0.937
EBV serology test				
Negative	47	271	1	
Positive	78	29	15.51(9.16-26.32)	<0.001
*Originally involved site*				
Paranasal structure	98			
Other sites	27			
*Tumor stage*				
I_E_	63			
II _E_	38			
III _E_	15			
IV _E_	9			

### IL-10, TNF, LTA, and CTLA-4 genotypes and haplotypes of NK/T lymphoma

The genotype and allele frequencies and the respective controls of the IL-10, TNF/LTA, and CTLA-4 gene regions in patients with NK/T lymphoma are listed in Table 3. All genotype frequencies were in Hardy–Weinberg equilibrium. Interestingly, the LTA +252 A > G polymorphism was significantly associated with the risk of developing NK/T lymphoma. Compared to common genotypes, the LTA +252 GA + AA genotype and A allele were associated with increased risk for NK/T-cell lymphoma (OR = 2.96, 95 % CI = 1.42–6.19, P = 0.004 for the GA + AA genotype; OR = 1.40, 95 % CI = 1.03–1.89, P = 0.03 for the A allele). After accounting for multiple comparisons, the LTA +252 GA + AA genotype remained significantly associated with NK/T-cell lymphoma. However, no statistical significance was noted in the overall risk of developing NK/T lymphoma for IL-10, TNF-α, and CTLA4 genotypes.

**Table 3:** Distribution of genotype and allele frequencies in with NK/T lymphoma patients and controls

Genotypes	Controls, n (%)	Cases, n (%)	OR (95 % CI)	*P* value
*IL-10 -3575*				
TT	278(93)	117(94)	1	
TA	22(7)	8(6)	0.86(0.37-1.99)	0.73
AA	0	0		
T	578(96)	242(97)	1	
A	22(4)	8(3)	0.87(0.38-1.98)	0.73
*IL-10 -1082*				
AA	237(79)	101(81)	1	
AG	60(20)	24(19)	0.94(0.55-1.59)	0.81
GG	3(1)	0(0)		
A	534(89)	226(90)	1	
G	66(11)	24(10)	0.86(0.52-1.41)	0.55
*IL-10 -819*				
CC	39(13)	9(7)	1	
CT	125(42)	59(47)	2.00(0.93-4.49)	0.08
TT	136(45)	57(46)	1.81(0.83-3.99)	0.14
C	203(34)	76(30)		
T	397(66)	174(70)	1.17(0.85-1.61)	0.33
*IL-10 -592*				
CC	38(13)	9(7)	1	
AC	124(41)	59(47)	2.00(0.91-4.43)	0.08
AA	138(46)	57(46)	1.74 (0.79-3.84)	0.17
C	200(33)	77(31)	1	
A	400(67)	173(69)	1.12(0.82-1.54)	0.47
*TNF-α -857*				
CC	239(79)	96(78)	1	
CT	56(19)	28(22)	1.25(0.75-2.08)	0.40
TT	5(1)	1(0)	0.50(0.06-4.32)	0.53
C	534(89)	220(88)	1	
T	66(11)	30(12)	1.10(0.69-1.75)	0.68
*TNF-α -308*				
GG	260(87)	115(92)	1	
AG	40(13)	10(8)	0.56 (0.27-1.17)	0.12
AA	0	0		
G	560(93)	240(96)	1	
A	40(7)	10(4)	0.58 (0.29-1.19)	0.14
*TNF-α -238*				
GG	267(89)	116(93)	1	
AG	32(11)	9(7)	0.65(0.30-1.40)	0.27
AA	1(0)	0(0)	-	
G	566(94)	241(96)	1	
A	34(6)	9(4)	0.62(0.29-1.32)	0.21
LTA +252				
GG	56(19)	9(7)	1	
GA	149(50)	71(57)	2.95(1.34-6.48)	0.01*
AA	95(31)	45(36)	2.97(1.39-6.33)	0.01*
GA + AA	244(81)	116(93)	2.96(1.42-6.19)	0.004*
G	261(44)	89(36)	1	
A	339(56)	161(64)	1.39(1.03-1.89)	0.03*
*CTLA-4 -1661*				
AA	216(72)	84(67)	1	
AG	78(26)	40(32)	1.32(0.84-2.08)	0.24
GG	6(2)	1(0)	0.43(0.05-3.61)	0.44
G	90(15)	42(17)	1	
A	510(85)	208(83)	0.87(0.59-1.30)	0.51
*CTLA-4 -318*				
CC	222(74)	88(70)	1	
CT	73(24)	36(29)	1.24(0.78-1.99)	0.36
TT	5(2)	1(0)	0.51(0.05-4.38)	0.54
C	517(86)	212(85)	1	
T	83(14)	38(15)	1.11(0.74-1.69)	0.60
*CTLA-4 + 49*				
AA	34(11)	8(6)	1	
GA	118(39)	60(48)	2.16(0.94-4.96)	0.07
GG	148(49)	57(46)	1.64(0.72-3.75)	0.24
A	186(31)	76(30)	1	
G	414(69)	174(70)	0.97(0.71-1.34)	0.86
CTLA-4 CT60				
AA	10(3)	3(2)	1	
GA	82(27)	32(26)	1.30(0.34-5.04)	0.70
GG	208(69)	90(72)	1.44(0.39-5.36)	0.58
A	102(17)	38(15)	1	
G	498(83)	212(85)	1.14(0.76-1.71)	0.52

^*^adjusted for age, sex

**Fig. 1:**
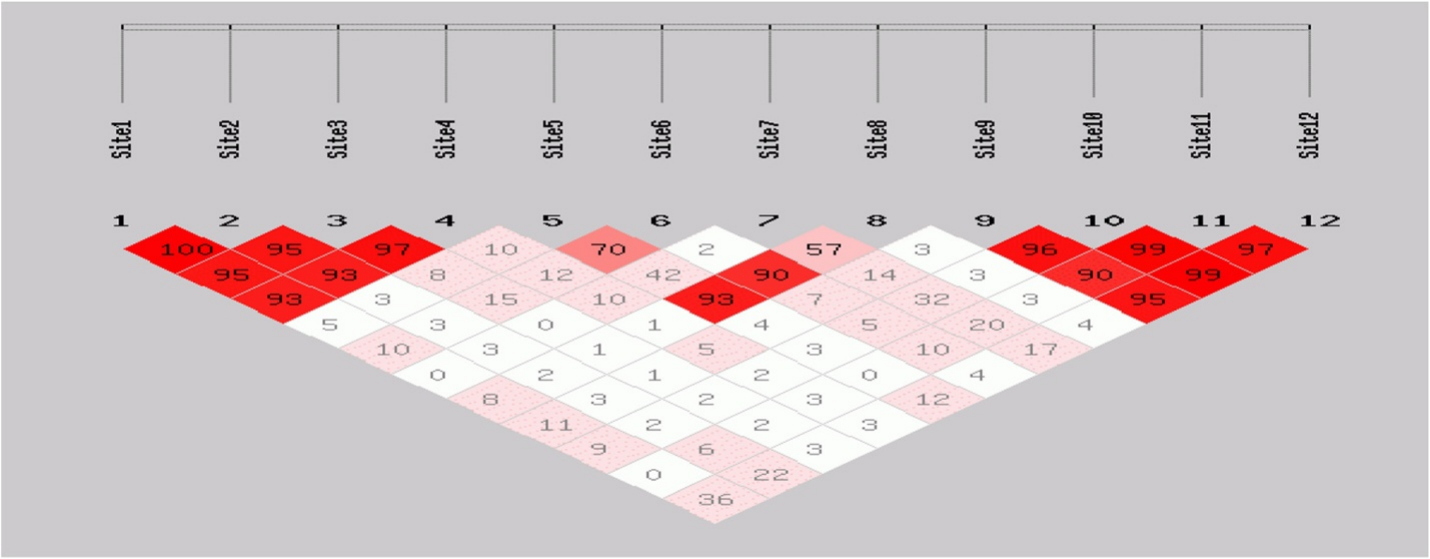
D’values for linkage disequilibrium between IL-10, TNF/LTA and CTLA-4 SNPs

Haplotype analyses were performed and the most common haplotype frequencies are shown in Table 4. Details of linkage disequilibrium tests (D’) are shown in Fig. 1. These analyses showed that the most common haplotype, C-G-G-A of TNF/LTA, had an increased risk of NK/T lymphoma (OR = 1.52, 95%CI = 1.13 ~ 2.04, P < 0.01), compared to carriers of the non-CGGA haplotype. No statistical difference was observed between IL-10 and CTLA-4 haplotypes and NK/T-cell lymphomas.

**Table 4:** Distribution of haplotype frequencies in NK/T-cell lymphoma patients and controls

haplotype	control	NK/T	OR(95%CI)	P
IL-10 -3575,-1082,-819,-592				
TATA	393(66)	173(69)	1	
TACC	136(23)	53(21)	0.89(0.62-1.27)	0.52
TGCC	40(7)	16(6)	0.91(0.49-1.67)	0.76
AGCC	22(4)	8(3)	0.83(0.36-1.89)	0.65
TNF-α-857,-308,-238,LTA-252				
CGGA	242(40)	127(51)	1.52(1.14-2.06)	0.005
Non-CGGA	358(60)	123(49)	1	
CGGG	221(37)	76(31)	1.01(0.72-1.39)	0.99
TGGA	65(11)	28(11)	1.25(0.77-2.04)	0.36
CAGG	38(6)	7(3)	0.54(0.23-1.3)	0.14
CTLA-4 -1661,-318,49,CT60				
ACGG	410(68)	170(68)	1	
ACAA	99(16)	38(15)	0.93(0.61-1.40)	0.71
GTAG	81(14)	38(15)	1.13(0.74-1.73)	0.57

### Stratification analysis by EBV infection of IL-10, TNF, LTA, and CTLA-4 genotypes and NK/T lymphoma

In this study, EBV infection was significantly higher for cases (62.4 %) than controls (9.7 %). Thus, the relationship between gene polymorphisms and NK/T lymphoma risk was further analyzed with respect to EBV serology. Except for the LTA + 252 genotype, no other genotypes were significantly associated with NK/T lymphoma risk. However, the LTA +252 GA + AA genotype was associated with an increased risk of NK/T lymphoma among EBV-positive populations (OR = 5.20, 95 % CI = 1.22–23.41, P = 0.03, Table 5). Nevertheless, we found no evidence of interaction between LTA +252 G > A polymorphism and EBV serology in relation to NK/T lymphoma risk (P interaction = 0.397). These data indicate that the LTA +252 GA + AA genotype likely influences the inflammatory response to EBV infection, which may ultimately alter the risk for developing NK/T lymphoma.

**Table 5:** Distribution of LTA +252 genotype frequencies in patients stratified by EBV status

LTA + 252G > A	Cases	Controls	OR (95 % CI)	*P* value
EBV positive				
GG	3	5	1	
AG + AA	75	24	5.20 (1.22-23.41)	0.03
EBV negative				
GG	6	51	1	
AG+ AA	41	220	1.6 1(0.63-3.92)	0.32

## Discussion

The genetic associations between IL-10, TNF/LTA, and CTLA-4 polymorphisms have been investigated extensively in many autoimmune diseases and malignancies, and have been confirmed for certain diseases. In lymphoma, IL-10 -3575A and TNF -308G increase the risk of DLBCL [11, 12], and CTLA-4 + 49 A > G increases the risk of MALT lymphoma [19]. In this study, our results showed that LTA +252 A > G polymorphism is associated with a 2.9-fold risk of NK/T-cell lymphoma, but other polymorphisms of the IL-10, TNF-α, and CTLA-4 genes are not. The most common haplotype, CGGA (TNF-α-857 C > T, -308 G > A, -238 G > A, LTA +252 A > G) conferred a 1.5-fold risk of NK/T-cell lymphoma. Furthermore, the LTA +252 GA + AA genotype was associated with an increased NK/T lymphoma risk among EBV–positive populations. Thus, our results suggest that the LTA +252 polymorphism may play an important role in the NK/T-cell lymphoma development, particularly in those who are EBV infected.

Previous studies have shown that LTA is necessary for the presence of NK cells in the spleen and LTA-/- mice have fewer splenic NK cells [20]. LTA signaling may be involved in the maturation and recruitment of NK cells and is required for NK cell activation [21]. In contrast, in NK cell-mediated anti-tumor activity, LTA contributes to tumor rejection by stimulating the host immune response [21]. For NK/T lymphoma, no role for LTA in NK/T-cell malignant transformation has been reported. Our study shows that individuals with the LTA +252 A allele have an increased tendency toward NK/T-cell lymphoma, suggesting that LTA deregulation caused by genetic polymorphisms may be affected by NK/T lymphoma pathogenesis. In our control group, we note that the LTA +252A and G allele frequencies were 0.56 and 0.44, respectively, similar to the frequencies in healthy Koreans (0.54 and 0.46, respectively) [22], but different from Caucasians of European descent (0.68 and 0.32, respectively) [9, 12]. This suggests that the LTA +252 A > G polymorphism varies among ethnic groups or geographical regions, and association of the LTA +252 A > G polymorphism with NK/T-cell lymphoma appears to vary by ethnicity.

The human LTA gene is located on chromosome 6p23-q12 and is closely linked to TNF-α, from which it is separated by about 1.2Kb. Our study also shows that the TNF/LTA haplotype CGGA (TNF-α -857C/-308G/-238G/LTA +252A) has a 1.5-fold increased risk of NK/T-cell lymphoma compared with those of non-CGGA types. The TNF/LTA haplotypes in most studies focused on the TNF-308 and LTA +252 loci, with significant associations between high-producer TNF-α- 308A/LTA +252G haplotypes and increased risk of DLBCL [9]. In contrast, our results suggest that TNF-α −308 G and LTA +252 A haplotypes increase the risk of NK/T-cell lymphoma. The TNF/LTA locus is located within the major histocompatibility complex (HLA) class III region. This region has many polymorphisms and regulates the immune response to infection and malignant transformation. Several studies have described substantial genetic variations in the HLA-DRB1 and LTA-TNF regions in Caucasians and Asians [23, 24], which may lead to different levels of NHL susceptibility. Fine mapping and functional studies of SNPs across this region will be required to determine whether the TNF-α–308 G > A and LTA +252 A > G SNPs constitute distinct susceptibility alleles or whether they are linked to other causal HLA loci.

In this study, EBV infection is commonly observed in nasal NK/T lymphoma, but its oncogenic mechanism remains unclear. Some studies have shown that EBV can be integrated into the host cell genome, causing lymphocyte immortalization [25, 26]. In addition, it has been suggested that EBV stimulates T lymphocytes, releasing a variety of cytokines such as TNF, interferon (IFN), and interleukin-1 (IL-1), which may lead to immune dysfunction [27]. Chronic inflammation induced by viral infection may result in complex interrelated degenerative and regenerative processes, promoting the accumulation of critical mutations in the host genome. This may be the reason why chronic inflammation is closely related to a number of cancers. The present study describes, for the first time, the significantly higher presence of the LTA +252 G > A genotype in NK/T lymphoma patients (OR = 2.9, 95 % CI = 1.42–6.19) and EBV-positive populations (OR = 5.2, 95 % CI = 1.22–23.41, P = 0.03). This leads us to infer that this allele combined with H. pylori infection may further increase the risk for developing NK/T-cell lymphoma.

LTA and TNF-α are key members of the TNF family, and are similar in gene structure, protein molecular structure, and biological function. However, they have many differences with respect to cellular origin and regulation of gene expression. Our results show that the LTA, but not the TNF gene, polymorphism serves as a genetic marker for NK/T-cell lymphoma, which suggests that subtle genetic differences may be involved in regulating different signaling pathways and leading to different pathogenesis among lymphoma subtypes.

## Conclusions

Our data suggest that the LTA +252 A > G polymorphism is associated with the risk of developing NK/T-cell lymphoma in a Chinese population, especially with EBV-positive NK/T-cell lymphoma.

## Abbreviations

NHL: Non-Hodgkin lymphoma
SNP: Single nucleotide polymorphism
NK: Natural killer
IL-10: Interleukin-10
TNF: Tumor necrosis factor
LTA: Lymphotoxin-α
CTLA-4: Cytotoxic T lymphocyte antigen 4
EBV: Epstein-Barr virus
DLBCL: Diffuse large B-cell lymphoma
PCR: Polymerase Chain Reaction
LDR: Ligase detection reactions
CI: Confidence intervals
OR: Odds ratios

## References

[1] Liu J, Song B, Fan T, Huang C, Xie C, Li J, Zhong W, Li S, Yu J (2011). Pathological and clinical characteristics of 1,248 non-Hodgkin's lymphomas from a regional cancer hospital in Shandong, China. Asian Pac J Cancer Prev.

[2] Hamidah NH, Shahrom S, Siti Aishah MA, Das S, Gendeh BS, Fadilah SA (2014). Nasal type NK/T-cell lymphoma - diagnosis and treatment difficulties. Clin Ter.

[3] Wang SS, Nieters A (2010). Unraveling the interactions between environmental factors and genetic polymorphisms in non-Hodgkin lymphoma risk. Expert Rev Anticancer Ther.

[4] Kim SY, Cho SG, Kim SW, Choi BO, Park KS, Lim J, Min CK, Kim YG, Lee JW, Min WS (2011). Pilot study of pegylated interferon alpha-2a treatment during chemo- and radiotherapy and post-remission maintenance in patients with EBV-positive extranodal NK/T cell lymphoma. Ann Hematol.

[5] Lan Q, Wang SS, Menashe I, Armstrong B, Zhang Y, Hartge P, Purdue MP, Holford TR, Morton LM, Kricker A, Cerhan JR, Grulich A, Cozen W, Zahm SH, Yeager M, Vajdic CM, Schenk M, Leaderer B, Yuenger J, Severson RK, Chatterjee N, Chanock SJ, Zheng T, Rothman N (2011). Genetic variation in Th1/Th2 pathway genes and risk of non-Hodgkin lymphoma: a pooled analysis of three population-based case-control studies. Br J Haematol.

[6] Hosgood HD, Purdue MP, Wang SS, Zheng T, Morton LM, Lan Q, Menashe I, Zhang Y, Cerhan JR, Grulich A, Cozen W, Yeager M, Holford TR, Vajdic CM, Davis S, Leaderer B, Kricker A, Schenk M, Zahm SH, Chatterjee N, Chanock SJ, Rothman N, Hartge P, Armstrong B (2011). A pooled analysis of three studies evaluating genetic variation in innate immunity genes and non-Hodgkin lymphoma risk. Br J Haematol.

[7] Skibola CF, Bracci PM, Nieters A, Brooks-Wilson A, de Sanjosé S, Hughes AM, Cerhan JR, Skibola DR, Purdue M, Kane E, Lan Q, Foretova L, Schenk M, Spinelli JJ, Slager SL, De Roos AJ, Smith MT, Roman E, Cozen W, Boffetta P, Kricker A, Zheng T, Lightfoot T, Cocco P, Benavente Y, Zhang Y, Hartge P, Linet MS, Becker N, Brennan P, Zhang L, Armstrong B, Smith A, Shiao R, Novak AJ, Maynadie M, Chanock SJ, Staines A, Holford TR, Holly EA, Rothman N, Wang SS (2010). Tumor necrosis factor (TNF) and lymphotoxin-alpha (LTA) polymorphisms and risk of non-Hodgkin lymphoma in the InterLymph Consortium. Am J Epidemiol.

[8] Cao C, Liu S, Lou SF, Liu T (2014). The +252A/G polymorphism in the Lymphotoxin-α gene and the risk of non-Hodgkin lymphoma: a meta-analysis. Eur Rev Med Pharmacol Sci.

[9] Domingo-Domènech E, Benavente Y, González-Barca E, Montalban C, Gumà J, Bosch R, Wang SS, Lan Q, Whitby D (2007). Fernández de Sevilla A, Rothman N, de Sanjosé S: Impact of interleukin-10 polymorphisms (-1082 and -3575) on the survival of patients with lymphoid neoplasms. Haematologica.

[10] Nocturne G, Boudaoud S, Miceli-Richard C, Viengchareun S, Lazure T, Nititham J, Taylor KE, Ma A, Busato F, Melki J, Lessard CJ, Sivils KL, Dubost JJ, Hachulla E, Mariette X (2013). Germline and somatic genetic variations of TNFAIP3 in lymphoma complicating primary Sjogren's syndrome. Blood.

[11] Nasiri H, Farajnia S, Rezamand A, Movassaghpour AA, Esmaeili HA, Monfaredan A, Mobarra N, Rahimifar N, Sahebi L, Farshdousti Hagh M (2013). Genetic Variations of Tumor Necrosis Factor -α-308 and Lymphtoxin-α + 252 in Non-Hodgkin Lymphoma and Acute Lymphoblastic Leukemia Patients. Iran J Basic Med Sci.

[12] Rothman N, Skibola CF, Wang SS, Morgan G, Lan Q, Smith MT, Spinelli JJ, Willett E, De Sanjose S, Cocco P, Berndt SI, Brennan P, Brooks-Wilson A, Wacholder S, Becker N, Hartge P, Zheng T, Roman E, Holly EA, Boffetta P, Armstrong B, Cozen W, Linet M, Bosch FX, Ennas MG, Holford TR, Gallagher RP, Rollinson S, Bracci PM, Cerhan JR, Whitby D, Moore PS, Leaderer B, Lai A, Spink C, Davis S, Bosch R, Scarpa A, Zhang Y, Severson RK, Yeager M, Chanock S, Nieters A (2006). Genetic variation in TNF and IL10 and risk of non-Hodgkin lymphoma: a report from the InterLymph Consortium. Lancet Oncol.

[13] Czarneski J, Lin YC, Chong S, McCarthy B, Fernandes H, Parker G, Mansour A, Huppi K, Marti GE, Raveche E (2004). Studies in NZB IL-10 knockout mice of the requirement of IL-10 for progression of B-cell lymphoma. Leukemia.

[14] Etemadi N, Webb A, Bankovacki A, Silke J, Nachbur U (2013). Progranulin does not inhibit TNF and lymphotoxin-α signalling through TNF receptor 1. Immunol Cell Biol.

[15] Waterhouse P, Penninger JM, Timms E, Wakeham A, Shahinian A, Lee KP, Thompson CB, Griesser H, Mak TW (1995). Lymphoproliferative disorders with early lethality in mice deficient in Ctla-4. Science.

[16] Ueda H, Howson JM, Esposito L, Heward J, Snook H, Chamberlain G, Rainbow DB, Hunter KM, Smith AN, Di Genova G, Herr MH, Dahlman I, Payne F, Smyth D, Lowe C, Twells RC, Howlett S, Healy B, Nutland S, Rance HE, Everett V, Smink LJ, Lam AC, Cordell HJ, Walker NM, Bordin C, Hulme J, Motzo C, Cucca F, Hess JF, Metzker ML, Rogers J, Gregory S, Allahabadia A, Nithiyananthan R, Tuomilehto-Wolf E, Tuomilehto J, Bingley P, Gillespie KM, Undlien DE, Rønningen KS, Guja C, Ionescu-Tîrgovişte C, Savage DA, Maxwell AP, Carson DJ, Patterson CC, Franklyn JA, Clayton DG, Peterson LB, Wicker LS, Todd JA, Gough SC (2003). Association of the T-cell regulatory gene CTLA4 with susceptibility to autoimmune disease. Nature.

[17] Karabon L, Pawlak-Adamska E, Tomkiewicz A, Jedynak A, Kielbinski M, Woszczyk D, Potoczek S, Jonkisz A, Kuliczkowski K, Frydecka I (2012). Variations in suppressor molecule ctla-4 gene are related to susceptibility to multiple myeloma in a polish population. Pathol Oncol Res.

[18] Khorshied MM, Gouda HM, Khorshid OM (2014). Association of cytotoxic T-lymphocyte antigen 4 genetic polymorphism, hepatitis C viral infection and B-cell non-Hodgkin lymphoma: an Egyptian study. Leuk Lymphoma.

[19] Cheng TY, Lin JT, Chen LT, Shun CT, Wang HP, Lin MT, Wang TE, Cheng AL, Wu MS (2006). Association of T-cell regulatory gene polymorphisms with susceptibility to gastric mucosa-associated lymphoid tissue lymphoma. J Clin Oncol.

[20] Wu Q, Sun Y, Wang J, Lin X, Wang Y, Pegg LE, Fütterer A, Pfeffer K, Fu YX (2001). Signal via lymphotoxin-beta R on bone marrow stromal cells is required for an early checkpoint of NK cell development. J Immunol.

[21] Ito D, Back TC, Shakhov AN, Wiltrout RH, Nedospasov SA (1999). Mice with a Targeted Mutation in Lymphotoxin-α Exhibit Enhanced Tumor Growth and Metastasis: Impaired NK Cell Development and Recruitment. J Immunol.

[22] Fugger L, Morling N, Ryder LP, Georgsen J, Jakobsen BK, Svejgaard A, Andersen V, Oxholm P, Karup Pedersen F, Friis J (1989). Nco I restriction fragment length polymorphism (RFLP) of the tumor necrosis factor (TNF alpha) region in four autoimmune diseases. Tissue Antigens.

[23] Price P, Bolitho P, Jaye A, Glasson M, Yindom LM, Sirugo G, Chase D, McDermid J, Whittle H (2003). A Gambian TNF haplotype matches the European HLA-A1, B8, DR3 and Chinese HLAA33, B58, DR3 haplotypes. Tissue Antigens.

[24] Ovsyannikova IG, Vierkant RA, Pankratz VS, Jacobson RM, Poland GA (2010). Extended LTA, TNF, LST1 and HLA gene haplotypes and their association with rubella vaccine-induced immunity. PLoS One.

[25] Amoli MM, Carthy D, Platt H, Ollier WE (2008). EBV Immortalization of human B lymphocytes separated from small volumes of cryo-preserved whole blood. Int J Epidemiol.

[26] Choi SM, Liu H, Chaudhari P, Kim Y, Cheng L, Feng J, Sharkis S, Ye Z, Jang YY (2011). Reprogramming of EBV-immortalized B-lymphocyte cell lines into induced pluripotent stem cells. Blood.

[27] Ohga S, Nomura A, Takada H, Tanaka T, Furuno K, Takahata Y, Kinukawa N, Fukushima N, Imai S, Hara T (2004). Dominant expression of interleukin-10 and transforming growth factor-beta genes in activated T-cells of chronic active Epstein-Barr virus infection. J Med Virol.

